# Complete plastome sequence of *Macaranga tanarius* (L.) Muell. Arg. (Euphorbiaceae): a fast-growing timber species

**DOI:** 10.1080/23802359.2021.1888332

**Published:** 2021-03-16

**Authors:** Jiang-Qu Tang, Xiao-Feng Zhang, Zhi-Xin Zhu, Hua-Feng Wang

**Affiliations:** Hainan Key Laboratory for Sustainable Utilization of Tropical Bioresources, College of Tropical Crops, Hainan University, Haikou, China

**Keywords:** *Macaranga tanarius*, Euphorbiaceae, plastome, plstome structure, phylogenetic

## Abstract

*Macaranga tanarius* (L.) Muell. Arg. is a tree species within Euphorbiaceae, which can be used for building timber and its extract can be used to treat diabetes. In this report, we describe the complete plastome sequence of *Macaranga tanarius*. The complete plastome of *Macaranga tanarius* (L.) Muell. Arg. is of 165,362 bp in length, and it is with typical plastome structure and gene content of angiosperm plastome, including two reverse repeat regions (IRs) of 27,503 bp, large single copy region (LSC) of 91,443 bp, and small single copy (SSC) region of 18,913 bp. The plastome contains 131 genes, including 85 protein coding genes, 38 tRNA genes, eight rRNA genes (i.e., 5S rRNA, 4.5S rRNA, 16S rRNA, and 23S rRNA). The total G/C content of *Macaranga tanarius* (L.) Muell. Arg.plastome is 35.6%. The complete plastome sequence is conducive to the development and utilization of Euphorbiaceae resources and the phylogenetic study.

## Introduction

*Macaranga tanarius* (L.) Muell. Arg. is a fast-growing multifunctional tree species ranging from four to fifteen meter. *M. tanarius* is distributed in Taiwan, Hong Kong and Guangdong Province of China. *M. tanarius* (L.) Muell. Arg. is a pioneer tree in reforestation projects and its leave extract can be used to treat diabetes. However, there is no report on complete plastome sequence of *M. tanarius* (L.) Muell. Arg. Therefore, it is of great significance to study the plastome of *M. tanarius* for its material development and utilization and systematic implications. In this report, we describe the complete plastome sequence of *Macaranga tanarius* (GenBank accession number: MW297079, this study) in order to promote the development and utilization of germplasm resources and provide useful genomic resources.

The samples for this study were collected from Ruili City, Yunnan Province, China (97.81°E, 24.16°N). The voucher sample (voucher code, RL0633) and its DNA were deposited in the Herbarium of China National GeneBank (code of herbarium: HCNGB).

The experimental process is followed Zhu et al. (2018). Approximately six Gb of clean data was assembled with plastome of *Ricinus communis* (JF937588.1) (Rivarola et al. 2011) using MITObim v1.8 (Le Petit queville, France, Hahn et al. 2013). Using Geneious r11.0.4 (biomatters Ltd., Auckland, New Zealand) to annotate the plastome of *Mallotus peltatus* (L.) Muell. Arg. (NC_047284.1).

The results show that the length of the plastome is 165,362 bp, which is with the typical quadrilateral structure of angiosperms, including two reverse repeats (IRs) of 27,503 bp, one large single copy (LSC) region of 91,443 bp and one small single copy (SSC) region of 18,913 bp. Plastome contains 131 genes, including 85 protein coding genes (six of which are repeated in IR), 38 tRNA genes (seven of which are repeated in IR), and eight rRNA genes (5S rRNA, 4.5 s rRNA, 16S rRNA, and 23S rRNA). The content of total G/C in the plastome is 35.6%. The corresponding values of LSC, SSC and IR are 33.1%, 30.0%, and 41.9%, respectively.

**Figure 1.**
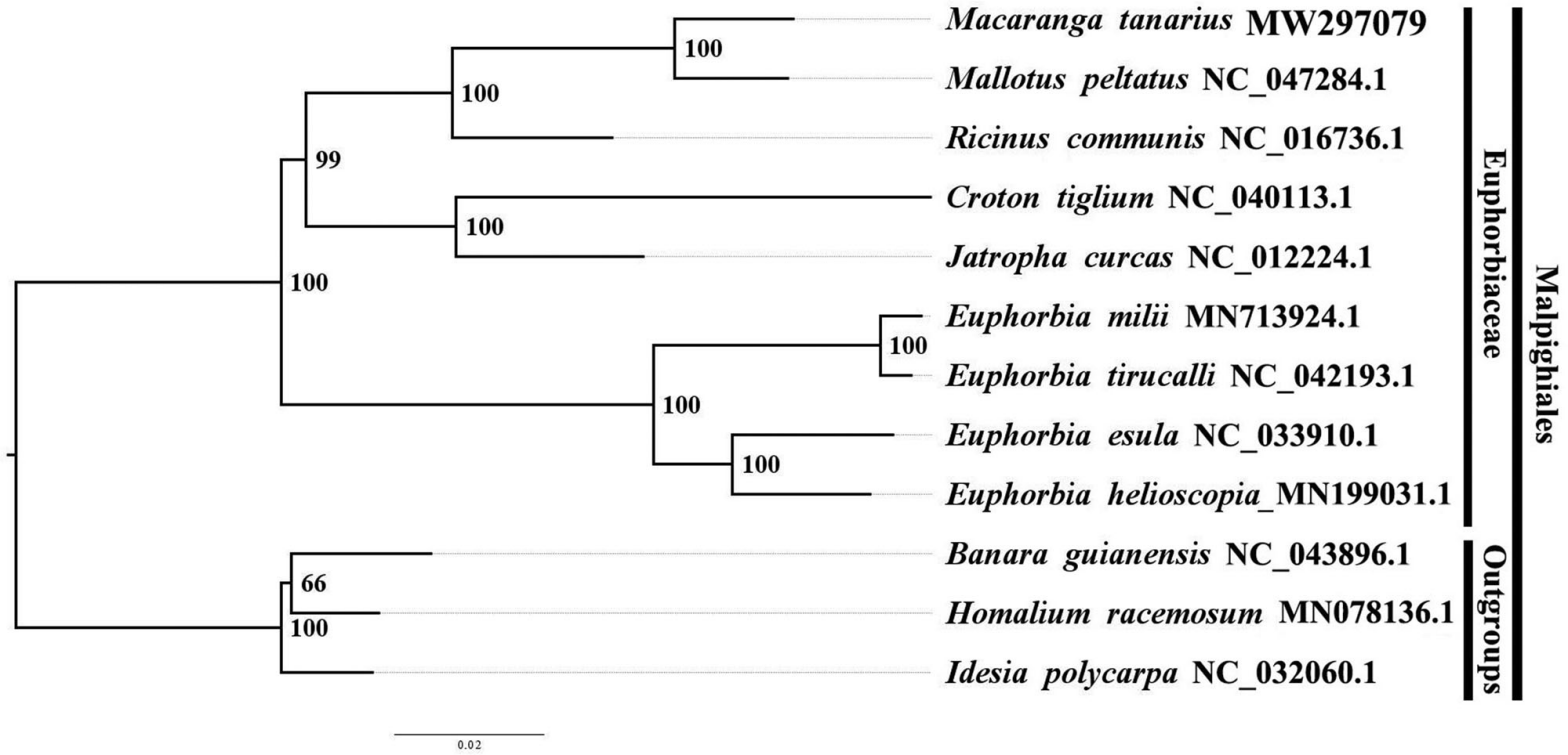
The best ML phylogeny recovered from 12 complete plastome sequences by RAxML. Accession numbers: *Macaranga tanarius* (GenBank accession number, MW297079, this study), *Mallotus peltatus*, NC_ 047284.1; *Ricinus communis*, NC_016736.1;*Croton tiglium,* NC_040113.1 *Jatropha curcas*, NC_ 012224.1; *Euphorbia milii*, MN713924.1; *Euphorbia tirucalli*, NC_042193.1; *Euphorbia esula*, NC_033910.1; *Euphorbia helioscopia*, MN_199031.1.three Outgroups: *Banara guianensis*, NC_ 043896.1; *Homalium racemosum*, MN_ 078136.1; *Idesia polycarpa*, NC_032060.1.

We used RAxML (Stamatakis 2006) to reconstruct the maximum likelihood (ML) phylogeny of eight published complete plastomes of Euphorbiaceae with 1,000 guiding sequences under the GTRGAMMAI substitution model, with *Banara guianensis* Aubl. NC_ 043896.1, *Homalium racemosum* Jacq. MN_078136.1 and *Idesia polycarpa* Maxim. NC_032060.1 as the outgroups. After reconstructing phylogenetic relationships of *M. tanarius* (L.) Muell. Arg. and published related taxa within Euphorbiaceae, we found that the phylogenetic relationships between *M. tanarius* (L.) Muell. Arg. and *Mallotus peltatus* (Geiseler) Müll. Arg. was closer than the relationships between *M. tanarius* (L.) Muell. Arg. and other taxa in Euphorbiaceae (Figure 1). It is beneficial to understand the systematic position of *M. tanarius* within Euphorbiaceae.

## Data Availability

The genome sequence data supporting the results of this study are publicly available on GenBank of NCBI (https://www.ncbi.nlm.nih.gov/) with registration number MW297079. The associated BioProject, SRA, and Bio-Sample numbers are PRJNA438407, SRS3260884, and SAMN08770805, respectively.
